# A Novel Instrumentation Circuit for Electrochemical Measurements

**DOI:** 10.3390/s120709687

**Published:** 2012-07-17

**Authors:** Li-Te Yin, Hung-Yu Wang, Yang-Chiuan Lin, Wen-Chung Huang

**Affiliations:** 1 Department of Optometry, Chung Hwa University of Medical Technology, Tainan 717, Taiwan; E-Mail: leaderyin@gmail.com; 2 Department of Electronic Engineering, National Kaohsiung University of Applied Science, Kaohsiung 807, Taiwan; E-Mail: jackyc_lin@compal.com; 3 General Education Center, Chung Hwa University of Medical Technology, Tainan 717, Taiwan; E-Mail: tom709@kimo.com

**Keywords:** instrumentation, biosensor, electrochemical measurement, multi-purpose

## Abstract

In this paper, a novel signal processing circuit which can be used for the measurement of H^+^ ion and urea concentration is presented. A potentiometric method is used to detect the concentrations of H^+^ ions and urea by using H^+^ ion-selective electrodes and urea electrodes, respectively. The experimental data shows that this measuring structure has a linear pH response for the concentration range within pH 2 and 12, and the dynamic range for urea concentration measurement is in the range of 0.25 to 64 mg/dL. The designed instrumentation circuit possesses a calibration function and it can be applied to different sensing electrodes for electrochemical analysis. It possesses the advantageous properties of being multi-purpose, easy calibration and low cost.

## 1. Introduction

The prototype of biosensors was first proposed by Clark and Lyon in 1962 [1]. The analytical method of detecting organisms actually exploits the molecular recognition between enzyme and acceptor. This concept involves placement of an enzyme in close proximity to an electrode surface, where the enzyme is able to catalyze a reaction. The analysis is based on the measurements of the consumption of an elective reactant (O_2_) and the production of an electroactive product (H_2_O_2_) [2]. The sensing mechanism of the biosensor is dependent on the biological specificity of the enzyme-catalyzed reaction and the selectivity of the ion-selective electrode, and hence, the characteristics of the biosensor are strongly related to the selectivity of the ion-selective electrodes. The enzyme electrode is a miniature chemical transducer which functions by combining an electrochemical procedure with immobilized enzyme activity. In 1967, Updike and Hicks used glucose oxidase immobilized on a gel to measure the concentration of glucose in biological solutions and in tissues *in vitro* [3]. From this moment on, many researches devoted to the development of biosensors, such as the O_2_, H_2_O_2_, H_2_, H^+^, NH_3_, CO_2_ electrodes and ion-sensitive field effect transistor (ISFET) [4]. An ISFET can be considered as a special type of the MOSFET without a metal or polysilicon gate, with the gate coated with a hydrogen ion-sensitive layer [5]. The gate of ISFET is directly exposed to the buffered solution to detect the concentration of hydrogen ion. The extended gate field effect transistor (EGFET) is another sensing structure which isolates the FET from the chemical environment.

Biosensors mainly composed of two parts. The first part is the sensing element which receives the input signal for the biological sensor. It can be the organism molecules, tissue or molecular recognition elements of individual cells. The other part is the electronic circuit which processes the quantified electronic signals from sensing element and outputs the processing result [6]. Therefore, the way to get accurate biological information quickly from sensing element and its processing circuit receive much attention of researchers [7,8]. Electrochemical sensors are widely utilized in many applications, such as disease diagnosis, food inspection and environmental monitoring, because of their fast reaction, high selectivity, high sensitivity, and simplicity [9].

In this study, based on the potentiometric method, an electronic instrumentation circuit is designed to detect the concentrations of H^+^ ions and urea by using H^+^ ion-selective electrodes and urea electrodes. The system performance for the H^+^ ions concentration detection can achieve the same accuracy as the commercial pH meter. The urea concentration detection using urea biosensors based on the measurement of H^+^ ion concentration possesses the dynamic range between 0.25 and 64 mg/dL. The workability of the sensing system is verified by measurement results.

## 2. Realization of Sensing Configuration

**Figure 1.**
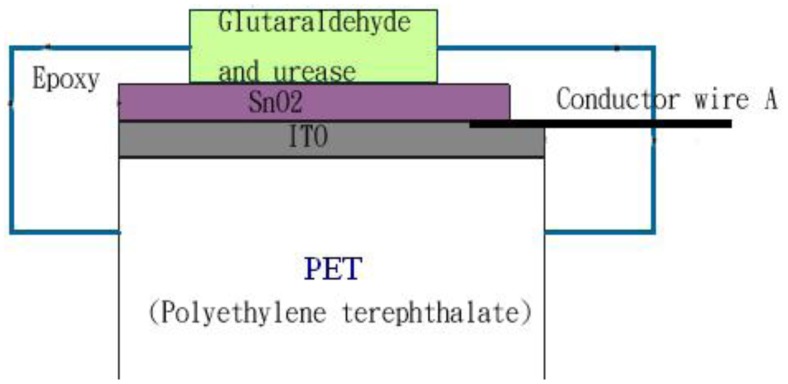
Electrode structure of ion-contact type.

**Figure 2.**
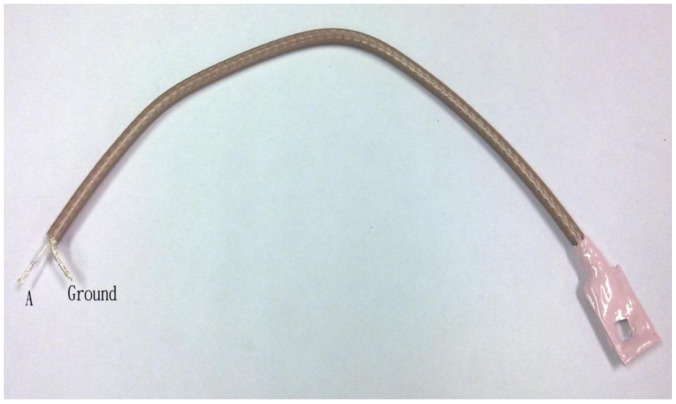
Practical pH electrode connected with a coaxial wire.

**Figure 3.**
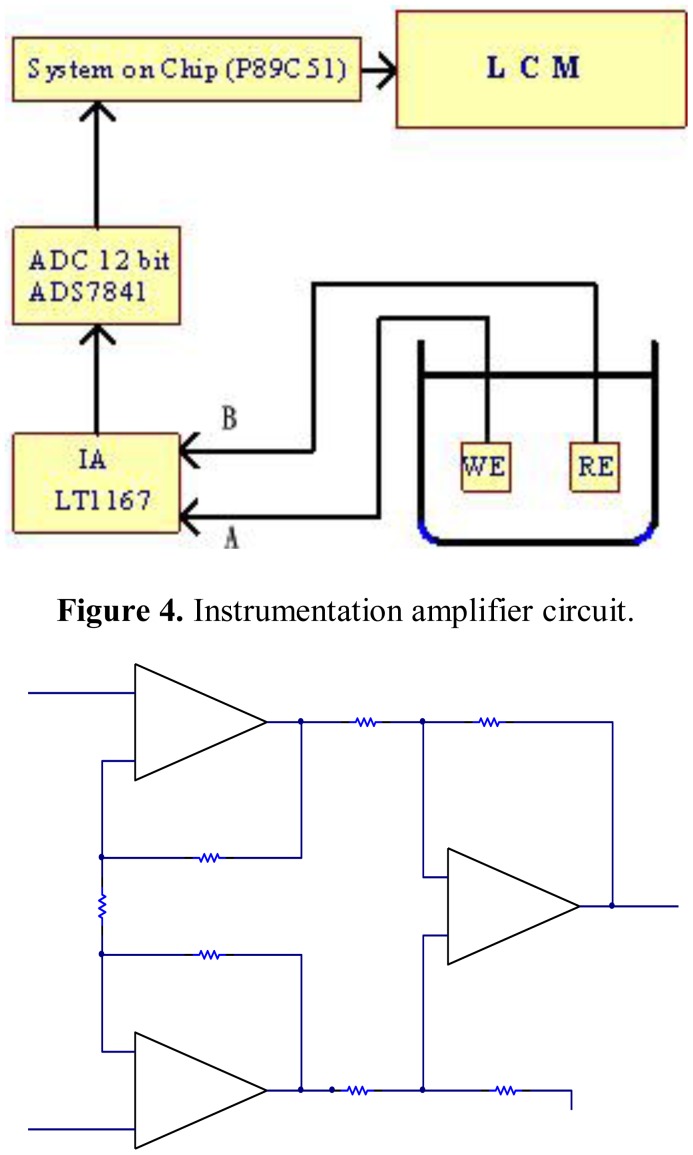
The system structure for potential measurement.

In this study, the used direct potential method is based on the measurement of potentials of electrode and the analysis of activity concentrations of ions employing the Nernst equation. The method usually uses indicating electrodes with ion-selective function. There are slight structural differences between the electrodes used and their general structure is shown in Figure 1. The SnO_2_/ITO/PET pH electrode in Figure 1 is based on a separated structure [10]. The SnO_2_ thin film is deposited at a thickness of 200 nm using sputtering [11]. Figure 2 shows the practical electrode connected with a coaxial wire to increase the immunity to external noise. For the measurement of the concentrations of H^+^ ions, the pH sensing area acting as working electrode (WE) and Ag/AgCl reference electrode (RE) were dipped into buffer solution and connected to the input terminals A and B of the designed instrumentation, as shown in Figure 3. Figure 3 also shows the proposed potential system structure used for the concentration measurement of hydrogen ion. The circuit mainly consists of an 8-bit microprocessor chip module (P89C51RB2HBA, Philips), an analog to digital converter (ADC), a liquid crystal display module (LCM) and a precision voltage amplifier. The voltage amplifier is implemented by an instrumentation amplifier (IA) to make good use of its characteristics of low-noise, high input impedance, low output impedance and tunable gain of the instrumentation amplifier. The commercially available pH meter usually set the zero potential which corresponds to pH 7. One unit change of pH value corresponds to the voltage change of about 59.1 mV. In theory, pH 14 to pH 0 will have the voltage from −413.7 mV to +413.7 mV.

**Figure 4.**
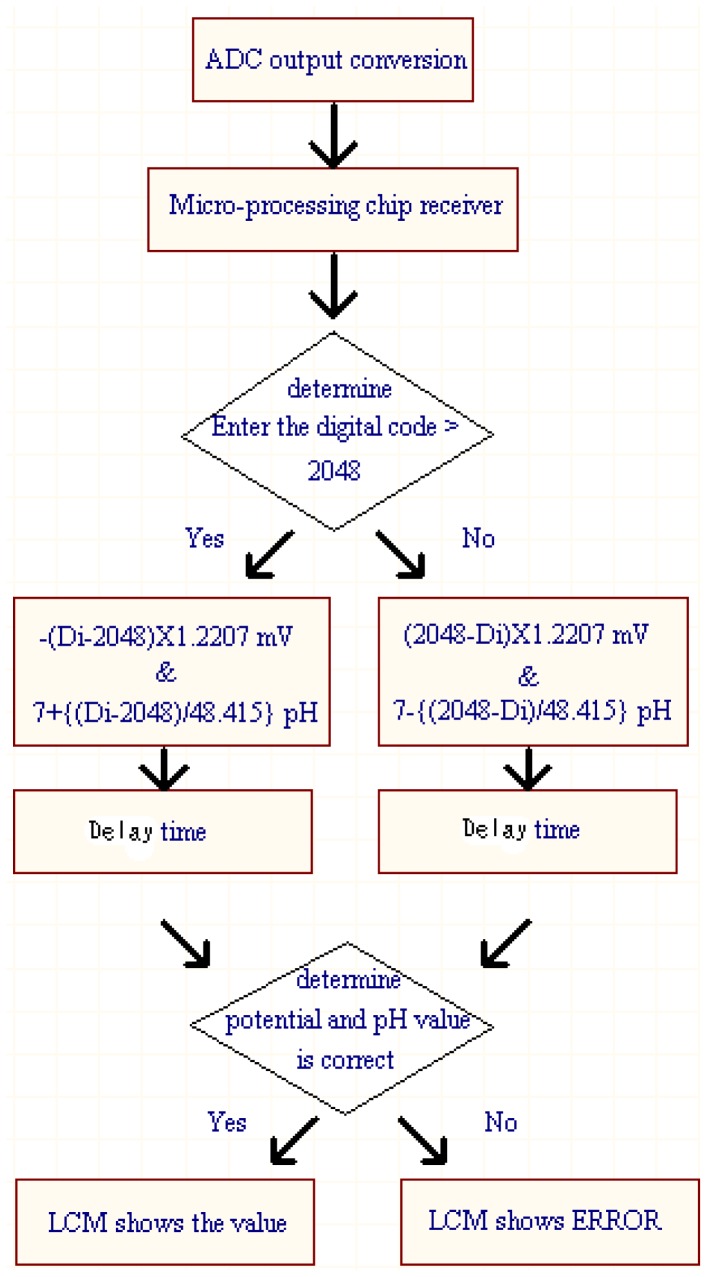
Instrumentation amplifier circuit.

For the proposed system in Figure 3, the ADC (ADS7841, Texas Instruments) is a single-supply chip, and the input voltage range of analog channel is a positive voltage between 0 V to 5 V. Thus the low-offset voltage and input bias current, high linearity and low gain error IA (LT1167, Linear Technology) with positive reference potential bias is used to construct the instrument, as shown in Figure 4. The positive reference potential bias can be obtained with a level-shift circuit. To calibrate the system and maximize the measurement range, the designed instrument has default setting that the output voltage of the IA for potential 2.5 V which corresponds to pH 7 of the buffer solution and code 2048 of ADC.

**Figure 5.**
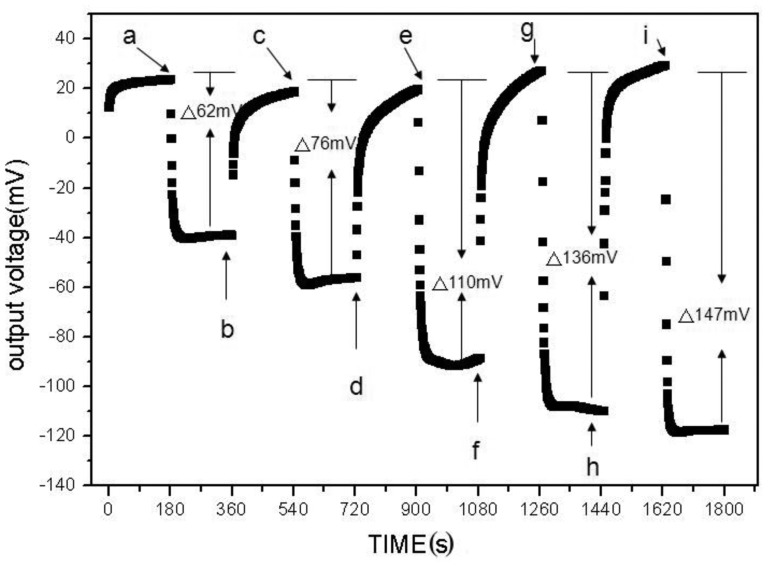
The internal operation of P89C51 for pH measurement.

The 12-bit ADC (ADS7841) is operated with a supply voltage of 5 V. Due to its input voltage range of 0 to 5 V, the minimum voltage step can be 5/212 V = 1.2207 mV. The default setting is that the change of per unit of pH value corresponds to 59.1 mV potential change, but this value is settable for our system. Therefore, the accuracy of measured pH value can achieve the resolution of about one digit after decimal point. It is enough for the measurement of general chemical laboratory. Figure 5 shows the internal program operation flow chart for P89C51 chip. For the operation procedure in Figure 5, the measured value is obtained using the output code of the ADC multiply by minimum voltage step (1.2207 mV). The pH value corresponding to its measured voltage value can be derived according to the procedure in Figure 5. An adjustable delay time is added to stabilize the displayed output values on LCM since the output codes of ADC (with conversion rate of 200 ksample/s) is refreshed too frequently. Then the measured value is checked and displayed on LCM.

According to the Nernst equation, it can be found that the measured voltage of a solution will be different at different temperatures. Therefore, the designed instrument has adjustable setting functions. That is, it has default setting that the 2.5 V output voltage of the IA corresponds to the measured voltage of calibrated solution of pH 7 and code 2048 of ADC. However, this corresponding code of ADC is settable. By inputting the program with the correct code of ADC (which is obtained by measuring the buffer solution of pH 7) from the COM port in designed electrochemical sensing instrumentation, the calibration can be attained. Therefore, the temperature calibration function can be achieved by this designed circuit. In addition, the designed program in P89C51 has the reset function. It adopts the mean of 50 output codes of ADC to process when the reset function is triggered.

Moreover, the urea biosensor circuit is constructed. The similar potentiometric method is used for the concentration measurement of urea solutions by using urea electrodes. The urea solution with higher concentration results in higher measured potentials according to:

(1) $$CO{\left(NH_{2}\right)}_{2}+3H_{2}O\to CO_{2}+2NH_{2}^{+}+2OH^{-}$$

**Figure 6.**
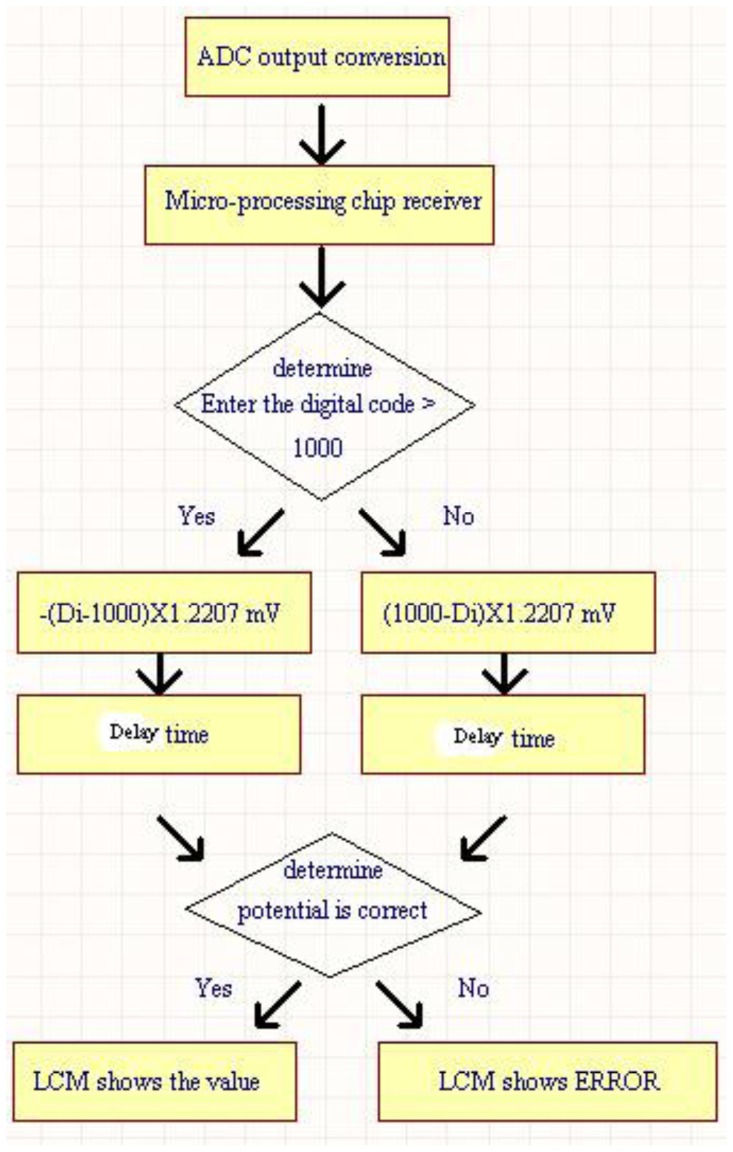
Measurement result of urea concentration.

The differences between the concentration measurement instrument for H^+^ ion and urea are the different sensing electrodes and the internal program of P89C51. For setting the instrumentation, the five urea solutions with concentrations of 8 mg/dL (a), 12 mg/dL (c), 16 mg/dL (e), 24 mg/dL (g) and 32 mg/dL (i) are used for calibration, as shown in Figure 6. It also shows the measured potential difference for different urea solutions. We can observe that the output voltages are increased with the higher concentration of urea solutions.

**Figure 7.**
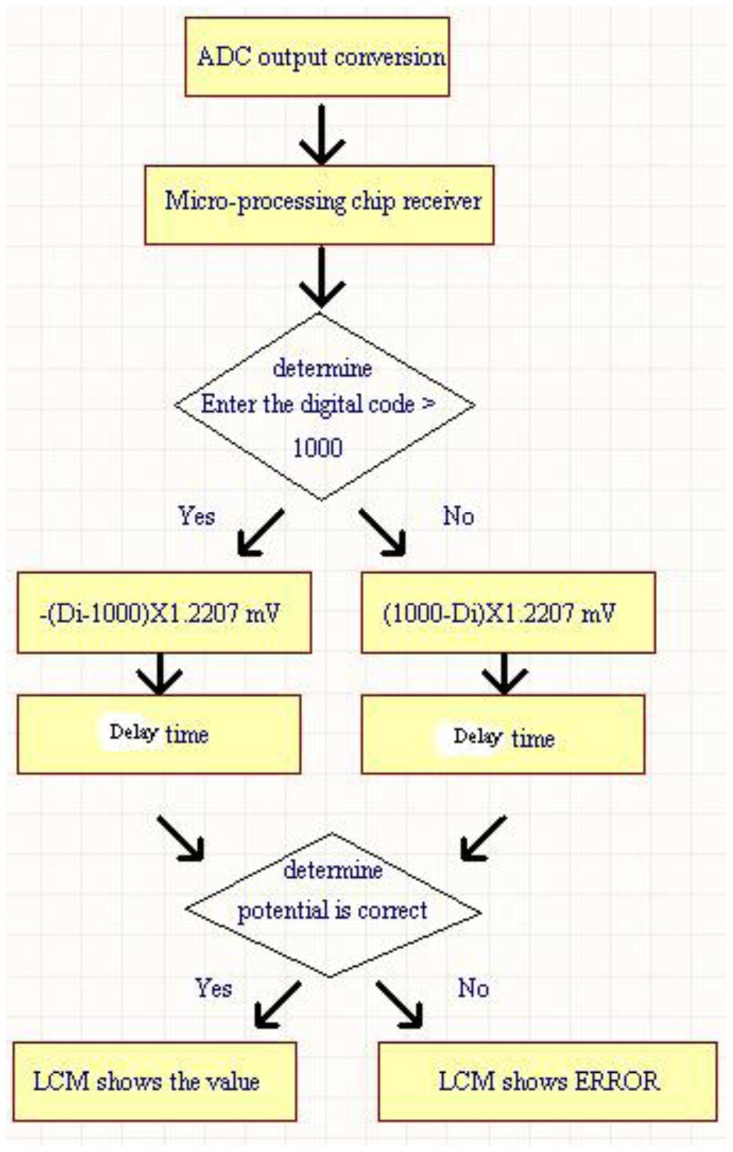
The internal operation of P89C51 for urea measurement.

As shown in Figure 7, the output voltage of 1.2207 V corresponds to output code 1000 of ADC of the instrument because the mentioned minimum voltage step is 1.2207 mV. The electrode is immersed in the phosphate buffer (PB) solution for resetting after each concentration measurement of different urea solutions. However, we observe that the restored voltage is not an exact 1.2207 V after the electrode is immersed in the PB solutions. To solve this problem, the microprocessor is programmed such that the default 1.2207 V which corresponds to output code 1000 of ADC is adjustable.

By inputting the microprocessor with the derived code after the electrode was immersed in the PB solution to replace the default code 1000 of ADC, the reset function is attained. It must be noted that the selected IA, ADC and microprocessor chips for the realization of this designed instrument are based on the considerations of appropriate accuracy, low cost and easy modification and debugging for experimental demands.

## 3. Experimental Results

**Figure 8.**
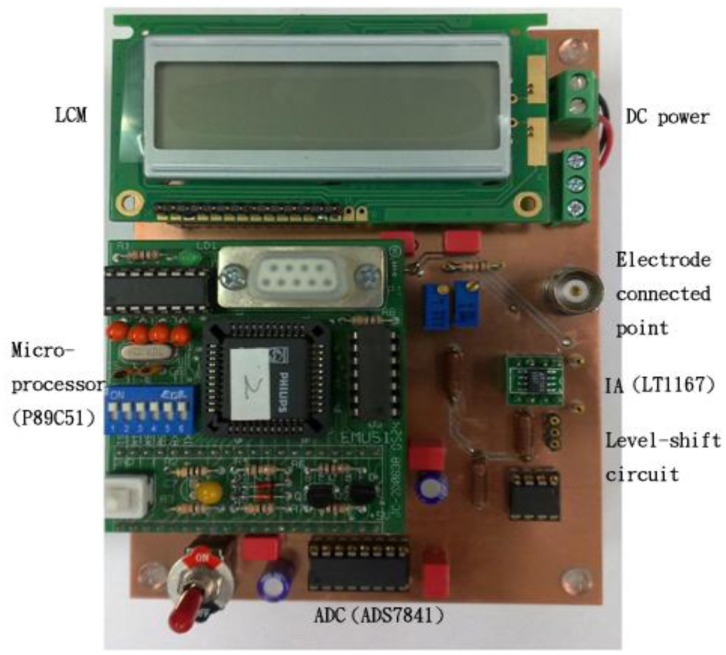
The designed electrochemical sensing instrumentation.

The designed circuit for practical measurement is shown in Figure 8. It is used to measure the concentrations of hydrogen ion and urea by using the H^+^ ion-selective electrodes and urea electrodes, respectively.

**Figure 9.**
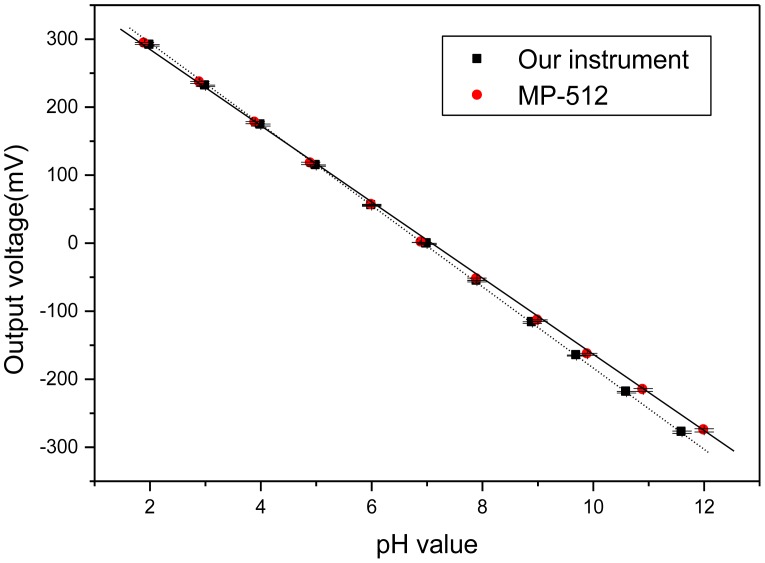
The plot of output voltage for different pH solutions.

To achieve better accuracy, three-point calibration was adopted for our experimental measurement. The buffer solutions of pH 7, 10, and 4 were used for calibration based on interpolation and extrapolation techniques. From the measured voltages for the buffer solutions of pH 7 and 10, we obtained the pH sensitivity of 53.8 mV/pH. Similarly, from the measured voltages for the buffer solutions of pH 7 and 4, we obtained the pH sensitivity of 58.7 mV/pH. These two values were inputted into the designed program in P89C51. Using the calibrated system to measure the pH values of different solutions, the result is shown in Figure 9. The circle symbol is obtained by using commercially available pH meter (MP-512) for concentration measurement of hydrogen ion. The square symbol is obtained using our designed instrument. The measurement results in Figure 9 confirm the feasibility of the designed system. The obtained standard deviations using our instrument and MP-512 are 4.16 and 1.56, respectively. The three-point calibration method can be further extended for multi-point calibration to achieve higher accuracy.

**Figure 10.**
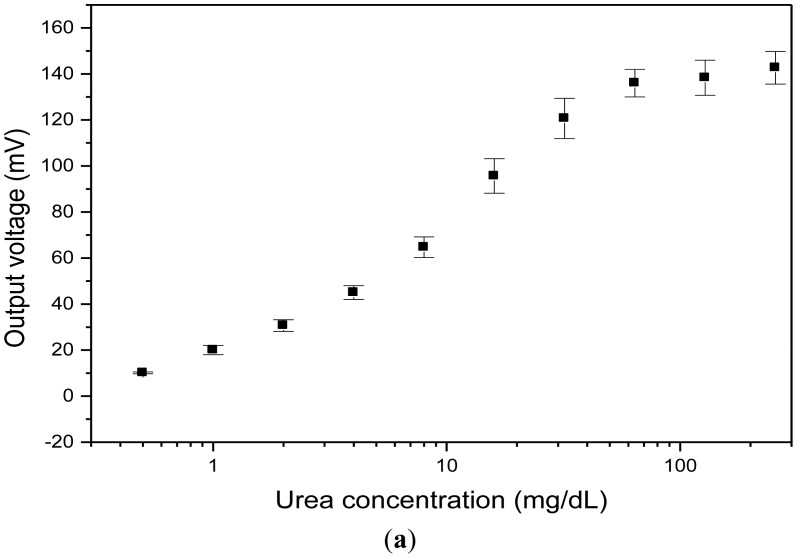

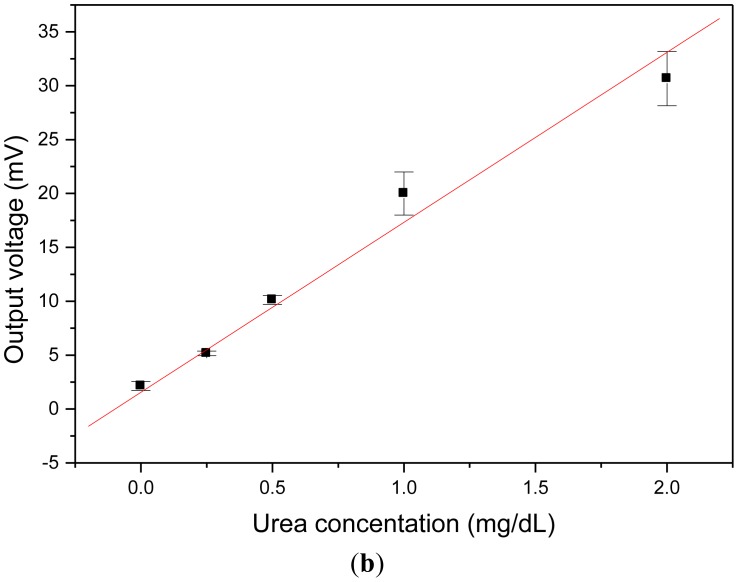
The output voltage for urea solutions with different concentrations. (**a**) Log scale; and (**b**) Linear scale.

The calibration curves of urea detection are shown in Figure 10. The dynamic range is between 0.25–64 mg/dL, as shown in Figure 10(a). In Figure 10(b), it can be observed that the linear range is between 0.25–2 mg/dL.

The square of the sample correlation coefficient is 0.97 and the standard deviation is 1.82. Although there seems to be few points in the linear range, the non-linear problem can be solved using interpolation to calculate the measured voltages with microprocessor chip. By the blank determination method [12], the average output value of 1.2 mV and standard deviation of 0.6 mV were obtained for the blank, as shown in Figure 10(b). The limit-of-detection (LOD) and limit-of-quantification (LOQ) in this study are 0.25 mg/dL and 0.5 mg/dL, respectively. The concentration of urea nitrogen of 1 mg/dL is equivalent to a concentration of urea of 2.14 mg/dL. For a normal person, the concentration of urea nitrogen is in the range of 6 mg/dL to 24 mg/dL. Thus the system with measured concentration range of the urea in Figure 10 can be used for basic biomedical examinations.

## 4. Conclusions

An electrochemical potential measurement instrument using a potentiometric method is designed in this paper. It is applied to the concentration measurements of pH value and urea by connecting hydrogen ion-selective electrodes and urea electrodes. The urea concentration is obtained by virtue of the measurement of hydrogen ion concentration of solution after the reaction of urea sensing electrode and urea solution. Its feasibility and accuracy are considered by practical measurements. In the same way, with different sensing electrodes, the instrument can be used for the electrochemical measurement of different objects by slight adjustment of internal program. The instrument has the advantageous properties of multi-purpose, easy calibration and low cost. It could be further improved on volume, such as the integrated realizations of IA and ADC or IA, ADC and P89C51 microprocessor, since the operational amplifiers are the common core components for IA and ADC and these analog devices could be implemented in a single chip [13].
